# Associations of fasting blood glucose and the triglyceride–glucose index with aggressive pathological features of papillary thyroid carcinoma

**DOI:** 10.3389/fendo.2026.1931124

**Published:** 2026-10-21

**Authors:** Xiang Xu, Kun Gong, Chengqian Li, Yonglu Hu, Cong Liu, Pei Guo, Wenjuan Zhao, Yangang Wang, Bingzi Dong

**Affiliations:** 1 Department of Cardiology, The Affiliated Hospital of Qingdao University, Qingdao, China; 2 Department of Endocrinology and Metabolism, The Affiliated Hospital of Qingdao University, Qingdao, China; 3 Department of Echocardiography, The Affiliated Hospital of Qingdao University, Qingdao, China; 4 Department of Surgical Intensive Care Unit, Yantai Yantaishan Hospital, Yantai, China; 5 Medical Records Management Center, The Affiliated Hospital of Qingdao University, Qingdao, China

**Keywords:** extracapsular extension, fasting blood glucose (FBG), lymph node metastasis, papillary thyroid carcinoma (PTC), triglyceride-glucose (TyG) index

## Abstract

**Background:**

The prevalence of papillary thyroid carcinoma (PTC) along with abnormal glucose-lipid metabolism and obesity is rising. Metabolic abnormalities may be associated with adverse pathological features of PTC.

**Objective:**

To evaluate the associations between metabolic indicators and the pathological features of PTC, and to assess the relationships of fasting blood glucose (FBG) and the triglyceride–glucose (TyG) index with PTC aggressiveness.

**Methods:**

Clinical data from patients with histologically confirmed PTC were analyzed. Logistic regression was used to examine the associations between metabolic indicators and adverse pathological features. Receiver operating characteristic (ROC) curves, DeLong’s test, continuous net reclassification improvement (NRI), and integrated discrimination improvement (IDI) were used to evaluate the incremental value of FBG and TyG. Internal validation was performed using bootstrap resampling.

**Results:**

PTC patients with diabetes were associated with tumor diameter >1 cm, extracapsular extension, lymph node metastasis, intermediate-to-high recurrence risk, and advanced TNM stage. Elevated FBG (≥6.1 mmol/L), BMI (≥25 kg/m²), TG and UA were associated with multiple unfavorable pathological characteristics. The highest TyG quartile was associated with extracapsular extension (OR = 2.103, P<0.05). The areas under the ROC curve (AUCs) for the baseline, FBG, and TyG models were 0.710, 0.738, and 0.722for extracapsular extension and 0.712, 0.727, and 0.714 for lymph node metastasis, respectively. TyG showed no clear incremental value for lymph node metastasis. Bootstrap-corrected values ranged from 0.698 to 0.722, with calibration slopes of 0.881–0.902.

**Conclusion:**

Elevated FBG, TG, UA, BMI, and TyG were associated with adverse pathological features of PTC. FBG was associated with extracapsular extension and lymph node metastasis, whereas TyG was associated with extracapsular extension but showed no clear incremental value for risk reclassification of lymph node metastasis.

## Introduction

In recent years, the incidence of papillary thyroid carcinoma (PTC) has increased rapidly (1). However, the etiology of PTC is complex, and its pathogenesis remains uncertain. Several studies have implicated that genetics (2), hormones (3), excessive iodine intake (4), ionizing radiation (5) and insulin resistance (6)are related to the occurrence of PTC. Currently, with the continuous improvement of living standards, the incidence of glucose and lipid metabolic disorders and obesity has also been increasing year by year (7). Studies have shown an association between hyperglycemia and cancer development, with diabetes mellitus (DM) increasing the risk of malignant tumors by 1.2- to 4.58-fold (8). Recent reports have shown that hyperglycemia and obesity can promote PTC development and increase the risk of extracapsular extension and advanced TNM stage (9). However, findings from other studies have been inconsistent, with some reporting no significant association (10). 

The mechanisms by which obesity and metabolic abnormalities affect PTC may involve insulin resistance (IR) (11), thyroid stimulating hormone (TSH) (12), adipokines (13, 14), inflammatory response (15). Among these, IR, as a core component of metabolic syndrome, contributes to the development and progression of PTC via different signaling pathways (6, 11). The hyperinsulinemic-euglycemic clamp technique, as the gold standard for diagnosing IR, is expensive and time-consuming in clinical practice. The triglyceride-glucose index (TyG) has gradually emerged as a promising alternative marker and reliable indicator of IR (16). It has been established that the TyG index is significantly associated with the risk of cardiometabolic abnormalities and mortality (17). Currently, accumulating evidence suggested that the TyG index could predict the risk of various non-metabolic diseases or non-cardiovascular events. A meta-analysis demonstrated that TyG serves as a significant predictor of the overall risk of solid tumors (18). Alkurt et al. revealed that TyG serves as a practical tool for identifying a high-risk population for PTC (19). However, to date, few studies have investigated the relationship between the TyG and PTC progression.

In this retrospective study, we investigated the role of metabolic indicators in PTC. We aimed to evaluate the associations of FBG and the TyG index with PTC invasion and metastasis.

## Materials and methods

### Study design

PTC patients who underwent thyroidectomy at the Affiliated Hospital of Qingdao University from June 2020 to June 2024 were enrolled, and clinical data were collected by experienced researchers. A total of 1038 PTC patients who met the inclusion criteria were included. The inclusion criteria were as follows (1): postoperative pathological confirmation of PTC (2); age > =18 years (3); complete clinical data availability. The exclusion criteria were (1): other types of thyroid cancer (2), presence of other malignant tumors, major organ diseases, or autoimmune diseases (3); preoperative use of medications affecting thyroid function, lipid profile, uric acid or BMI (4); combination with distant metastases (5); BMI <18.5 kg/m² (6); incomplete laboratory or pathological information. This cross-sectional study was conducted according to the Strengthening the Reporting of Observational Studies in Epidemiology (STROBE) statement. The study was performed in accordance with the Declaration of Helsinki and was approved by the ethics committee of The Affiliated Hospital of Qingdao University (Ethics number QYFYWZLL27740).

### Clinical characteristics and biochemical measurements

Patients’ clinical data, serologic results, and imaging findings were collected. On the day before blood collection, participants were instructed to avoid high-fat and high-sugar foods and alcohol. Blood samples were collected from participants after fasting and abstained from smoking for 10 h. FBG was measured by hexokinase method (Roche kit), TG, TC, LDL-C, HDL-C, UA were measured by enzyme colorimetric method (Roche kit); serum TSH was measured by electrochemiluminescence immunoassay (Roche kit). Height and weight were measured in the morning after an overnight fast, and BMI was calculated as weight (kg)/height squared (m²). According to the World Health Organization classification, BMI ≥25 kg/m² was defined as overweight or obesity. T2DM was diagnosed according to the 2015 American Diabetes Association criteria, and FBG ≥6.1 mmol/L was classified as elevated. The TyG index was calculated as ln[TG (mg/dL) × FBG (mg/dL)/2]. An upper reference limit of 9.2 for TyG was derived using the upper reference limits for TG (1.92 mmol/L) and FBG (6.1 mmol/L). BMI and the metabolic indicators were dichotomized according to their respective reference limits, HDL-C was categorized using its lower reference limit, whereas all other indicators were categorized using their upper reference limits. TyG was additionally analyzed by quartiles. The baseline model for extracapsular extension included age, sex, TSH, BMI, TC, LDL-C, HDL-C, UA, number and distribution of primary tumor foci, and maximum primary tumor diameter. The baseline model for lymph node metastasis additionally included extracapsular extension. The FBG and TyG models were constructed by adding FBG or TyG, respectively, to the corresponding baseline model.

### Clinicopathological and imaging measurements

Clinicopathological data were collected through the hospital database and included the following parameters: maximum diameter of primary tumor focus, number of primary tumor foci (unifocal/multifocal), distribution of tumor foci (unilateral/bilateral), extracapsular extension, lymph node metastasis. Lymph node and distant metastases were assessed based on postoperative histopathology, fine-needle aspiration, iodine-131 whole-body scintigraphy (¹³¹I-WBS), and single-photon emission computed tomography/computed tomography (SPECT/CT) imaging. Extracapsular extension (ECE; local invasion), cervical lymph node metastasis (LNM; regional spread), initial recurrence-risk category, and TNM stage (risk of clinical progression) were considered study outcomes. ECE and LNM were used as the principal measures of PTC aggressive features, and the associations of metabolic abnormalities with these outcomes were examined in detail. Recurrence risk was stratified according to 2015 ATA guideline (20) and TNM stage was assessed according to the 8^th^ edition of the American Joint Committee on Cancer (AJCC) TNM staging system (21).

### Statistical methods

Statistical analyses were performed using SPSS version 24.0 and R software. Continuous variables are presented as the mean ± standard deviation or median (interquartile range), according to their distributions, and were compared using the independent-samples *t* test or Mann-Whitney *U* test, as appropriate. Categorical variables are presented as numbers (percentages) and were compared using the chi-square test or Fisher’s exact test. Multivariable binary logistic regression was used to evaluate the associations of metabolic indicators with aggressive pathological features of PTC, and results are reported as odds ratios (ORs) with 95% confidence intervals (CIs). ROC curve analysis was used to assess model discrimination, with calculation of the AUC and 95% CI. Pairwise DeLong tests were used to compare AUCs from correlated ROC curves, and continuous net reclassification improvement (NRI) and integrated discrimination improvement (IDI) were used to assess changes in discrimination and risk classification after adding FBG or TyG. Internal validation was performed using 1,000 bootstrap resamples to estimate optimism-corrected AUCs, Brier scores, and calibration slopes. All tests were two-sided, and P<0.05 was considered statistically significant.

## Results

### Metabolic indicators and clinicopathologic characteristics of PTC patients

A total of 1038 PTC patients were enrolled. Among them, 244 (23.51%) were male and 794 (76.49%) were female, with a male-to-female ratio of 1:3.25. Their ages ranged from 20 to 83 years, with a mean age of 45.71 ± 12.09 years. In addition, 102 patients (9.83%) were combined with T2DM, with a mean diabetes duration of 6.13 ± 5.14 years. The details of metabolic and clinicopathologic characteristics of PTC patients are presented in **Supplementary Table 1**.

### Association of DM with clinicopathological features and metabolic indicators in PTC patients

Among 1038 PTC patients, 102 (9.83%) had T2DM and 936 (90.17%) did not. Comparison of metabolic indicators between the two groups showed that the proportion of male patients and the levels of FBG, TG, BMI, TyG were higher in DM group (all P<0.05), whereas HDL-C was lower (P<0.05) (**Table 1**). Regarding clinicopathological characteristics, the DM group showed a higher proportion of primary tumor foci with a maximum diameter of >1 cm, extracapsular extension, lymph node metastasis, intermediate-to-high risk stratification for recurrence, and advanced TNM stage (III/IV) (all P<0.05) (**Table 1**).

**Table 1:** Comparison of metabolic indicators and clinicopathological characteristics between PTC combined with DM and non-DM groups.

Variable	Non-DM 936 (90.17%)	DM 102 (9.83%)	P
Age(year)	45.53 ± 12.08	47.33 ± 12.12	0.132
Gender (male, %)	210 (22.44%)	34 (33.33%)	0.014*
Metabolic indicators			
BMI (kg/m2)	24.84 (22.96-27.34)	26.37 (24.22-28.35)	<0.001*
FBG	4.97 (4.58-5.45)	8.48 (7.64-9.65)	<0.001*
TG	1.02 (0.72-1.55)	1.22 (0.80-1.64)	0.047*
TC	4.92 (4.32-5.70)	4.90 (4.45-5.50)	0.977
LDL-C	2.83 (2.34-3.36)	2.81 (2.44-3.44)	0.571
HDL-C	1.41 (1.20-1.65)	1.31 (1.10-1.53)	0.009*
UA	286.00 (243.00-339.00)	295.30 (249.58-360.15)	0.320
TyG	8.34 (7.94-8.75)	9.05 (8.53-9.39)	<0.001*
Clinicopathological characteristics			
Number of primary tumor foci (Multifocal, %)	313 (33.44%)	39 (38.24%)	0.331
Distribution of tumor foci (Bilateral, %)	233 (24.89%)	31 (30.39)	0.226
Maximum diameter of primary tumor foci (>1cm,%)	363 (38.78%)	52 (50.98%)	0.017*
Extracapsular extension (Yes, %)	222 (23.72%)	48 (47.06%)	<0.001*
Lymph node metastasis (Yes, %)	427 (45.62%)	73 (71.57%)	<0.001*
Recurrence risk stratification (Intermediate-high risk, %)	517 (55.24%)	81 (79.41%)	<0.001*
Advanced TNM stage (III -IV, %)	50 (5.34%)	13 (12.75%)	0.003*

DM, diabetes mellitus; BMI, body mass index; FBG, fasting blood glucose; TG, triglyceride; TC, total cholesterol; LDLC, low-density lipoprotein cholesterol; HDLC, high-density lipoprotein cholesterol; UA, uric acid; TyG, triglyceride-glucose index; TNM, tumor-node-metastasis staging. * indicates P<0.05.

To examine the adjusted associations between DM and the clinicopathological characteristics of PTC, binary logistic regression was performed with adjustment for age, sex, and thyroid-stimulating hormone (TSH). The results showed that PTC patients with DM had an increased risk of primary tumor foci with a maximum diameter>1cm, extracapsular extension, lymph node metastasis, intermediate-to-high risk stratification for recurrence, and advanced TNM stage (III/IV), with odds ratios (ORs) of 1.618, 2.867, 3.090, 3.157, and 2.313, respectively (all P<0.05) (**Table 2**).

**Table 2:** Adjusted associations between DM and clinicopathological characteristics of PTC patients.

Clinicopathological characteristics	OR	95%CI	P
Maximum diameter of primary tumor foci (>1cm)	1.618	1.072-2.442	0.022
Extracapsular extension	2.867	1.884-4.363	<0.001
Lymph node metastasis	3.090	1.955-4.884	<0.001
Recurrence risk stratification (Intermediate-to-high risk, %)	3.157	1.912-5.214	<0.001
Advanced TNM stage (III -IV, %)	2.313	1.088-4.917	0.029

### Relationship between metabolic indicators and the risk of clinicopathological characteristics in PTC patients

To further explore the influence of each metabolic indicators (FBG, TG, TC, LDL, HDL-C, UA and BMI) on pathological characteristics, binary logistic regression analysis was performed. Sex, age and TSH level were also included in the regression model. The results showed that, compared with normal FBG group (FBG<6.1mmol/L), the elevated FBG group (≥6.1 mmol/L) had an increased risk of multifocal primary tumor foci, bilateral tumor foci, maximum diameter of primary tumor foci >1 cm, extracapsular extension, lymph node metastasis, intermediate-to-high risk stratification for recurrence, and advanced TNM stage (III/IV) (all P<0.05), with ORs of 1.412, 1.535, 1.509, 3.163, 2.320, 2.803, and 2.139, respectively. The group with elevated TG (> 1.92 mmol/L) showed an increased risk of extracapsular extension (P<0.05, OR = 1.611). Elevated UA (>420 umol/L) was associated with an increased risk of extracapsular extension and intermediate-to-high risk stratification for recurrence (both P<0.05), with ORs of 2.940 and 2.156, respectively. Furthermore, patients with BMI≥25kg/m^2^ had an increased risk of multifocal tumor foci, maximum diameter of foci >1 cm, extracapsular extension, lymph node metastasis, intermediate-to-high risk stratification for recurrence, with ORs of 1.302, 1.577, 1.692, 1.302 and 1.855, respectively (all P<0.05). No significant differences were observed for the remaining metabolic indicators (all P>0.05) (**Table 3**).

**Table 3:** Relationship between metabolic indicators and pathological characteristics in PTC patients.

Metabolic indicators	Multifocal	Bilateral	Maximum diameter of primary tumor foci>1cm	Extracapsular extension	Lymph node metastasis	Intermediate-to-high risk stratification for recurrence	Advanced TNM stage (III -IV%)
FBG	1.412* (1.022-1.950)	1.535* (1.088-2.164)	1.509* (1.101-2.067)	3.163* (2.269-4.410)	2.320* (1.668-3.228)	2.803* (1.964-4.001)	2.139* (1.180-3.878)
TG	1.201 (0.843-1.711)	1.218 (0.830-1.787)	0.967 (0.684-1.366)	1.611* (1.119-2.318)	0.882 (0.625-1.246)	1.025 (0.725-1.451)	0.659 (0.300-1.447)
TC	1.008 (0.750-1.357)	1.064 (0.772-1.466)	0.953 (0.715-1.271)	0.885 (0.639-1.225)	0.950 (0.714-1.264)	0.979 (0.736-1.302)	1.265 (0.709-2.257)
LDL	1.008 (0.767-1.324)	0.993 (0.738-1.336)	0.964 (0.741-1.256)	1.122 (0.837-1.504)	0.979 (0.753-1.272)	1.049 (0.806-1.365)	1.183 (0.668-2.092)
HDL-C	1.010 (0.183-5.579)	0.615 (0.071-5.331)	3.052 (0.554-16.808)	1.552 (0.279-8.648)	0.912 (0.177-4.691)	1.345 (0.241-7.504)	12.017 (0.439-329.723)
UA	1.010 (0.587-1.739)	0.965 (0.525-1.772)	1.096 (0.657-1.827)	2.940* (1.727-5.005)	1.516 (0.895-2.567)	2.156* (1.203-3.864)	2.142 (0.599-7.661)
BMI	1.302* (1.002-1.692)	1.249 (0.940-1.661)	1.577* (1.224-2.031)	1.692* (1.274-2.247)	1.302* (1.002-1.692)	1.855* (1.434-2.400)	1.297 (0.733-2.293)

Data were presented as OR (95%CI).

Gender, Age, TSH level were adjusted in logistic regression model.

FBG, TG, TC, LDL-C, UA values were grouped by the upper limits of normal value, HDL-C was grouped by the lower limits of normal value. *indicates P < 0.05

### Comparison of pathological characteristics across TyG quartiles in PTC patients

The comparison of pathological characteristics across the four TyG quartiles is presented in **Table 4**. The results showed that, among the four quartiles of TyG, significant differences were observed in the rate of extracapsular extension and advanced (III/IV) TNM stage. The number of patients with extracapsular extension in Q4 of TyG was higher than that in the Q1, Q2 and Q3 groups (all p < 0.05). Similarly, more patients with advanced TNM stage (III/IV) were found in Q3 and Q4 group of TyG than in Q1 and Q2 groups. Further logistic regression analysis showed that the risk of extracapsular extension was increased in Q4 of TyG compared with the Q1group, with an OR of 2.103 (P<0.05) (**Table 4**).

**Table 4:** Comparison of pathological characteristics by quartiles of TyG in PTC patients.

Groups	Multifocal	Bilateral	Maximum diameter of primary tumor foci>1cm	Extracapsular extension	Lymph node metastasis	Intermediate-to-high risk stratification for recurrence	Advanced TNM stage (III -IV%)
TyG (Q1)	84 (32.1%)	68 (26.0%)	100 (38.2%)	55 (21.0%)	129 (49.2%)	148(56.5%)	8(3.1%)
TyG (Q2)	93 (36.6%)	65 (25.6%)	100 (39.4%)	53 (20.9%)	123 (48.4%)	141(55.5%)	9(3.5%)
TyG (Q3)	87 (33.1%)	66 (25.1%)	105 (39.9%)	71 (27.0%)	119 (45.2%)	151(57.4%)	22(8.4%)^bd^
TyG (Q4)	88 (34.0%)	65 (25.1%)	110 (42.5%)	91 (35.1%)^cef^	129 (49.8%)	158 (61.0%)	24(9.3%)^ce^
TyG (Q1, Q4)							
OR (95%CI)				2.103* (1.397-3.164)			1.391 (0.564-3.427)

^a-c^indicate comparisons of Q2, Q3 and Q4 with Q1 respectively; ^d, e^indicate comparisons of Q3 and Q4 with Q2 respectively; ^f^indicates comparison of Q3 with Q4. All P < 0.05.

Adjusted for age, gender, TSH, *P<0.05 compared to Q1

### Multivariate analysis of FBG and TyG on PTC aggressive features

To further clarify the effect of FBG on PTC aggressive features, logistic regression analysis was performed. Age, sex, TSH, BMI, TG, TC, LDL-C, HDL-C and uric acid were included as adjustment factor. The results showed that elevated FBG (≥6.1 mmol/L) was associated with higher odds of extracapsular extension and lymph node metastasis (both P< 0.05), with ORs of 2.965 and 2.516 respectively. Similarly, after adjustment for age, sex, TSH, BMI, TC, LDL-C, HDL-C and UA, elevated TyG was found to be positively associated with the extracapsular extension (OR = 2.296, P < 0.05). Following further adjustment for the number and distribution of tumor foci and maximum primary tumor diameter, with additional adjustment for extracapsular extension in the lymph node metastasis analysis, elevated FBG remained associated with extracapsular extension (OR = 2.78, 95% CI: 1.941-3.989, P<0.05) and lymph node metastasis (OR = 2.034, 95% CI: 1.413-2.929, P<0.05). Elevated TyG remained associated with extracapsular extension (OR = 2.337, 95% CI: 1.524-3.583, P<0.05) (**Table 5**).

**Table 5:** Logistic regression analysis of the associations of FBG and TyG with aggressive pathological features of PTC.

Group	FBG		TyG
	Extracapsular extension	Lymph node metastasis	Extracapsular extension
Un-adjusted	3.069 (2.209-4.265) *	2.170 (1.568-3.004) *	2.455 (1.689-3.568) *
Model 1	3.098 (2.215-4.333) *	2.390 (1.703-3.353) *	2.519 (1.718-3.694) *
Model 2	3.068 (2.191-4.296) *	2.380 (1.692-3.347) *	2.359 (1.602-3.476) *
Model 3	2.996 (2.132-4.210) *	2.508 (1.773-2.854) *	2.257 (1.517-3.356) *
Model 4	2.965 (2.105-4.178) *	2.516 (1.774-3.567) *	2.296 (1.493,3.530) *
Model 5	2.78 (1.941-3.989)*	2.034 (1.413-2.929)*	2.337 (1.524-3.583)*

Data were presented as OR (95%CI), *indicates p<0.05.

FBG, TyG values were grouped by the upper limits of normal value.

Model 1: adjusted for age, gender;

Model 2: adjusted for age, gender, TSH;

Model 3: adjusted for age, gender, TSH, BMI;

FBG Model 4: adjusted for age, gender, TSH, BMI, TG, TC, LDL-C, HDL-C, uric acid

TyG model 4: adjusted for age, gender, TSH, BMI, TC, LDL-C, HDL-C, uric acid

FBG Model 5 (Extracapsular extension): adjusted for age, gender, TSH, BMI, TG, TC, LDL-C, HDL-C, uric acid, Number of primary tumor foci, Distribution of tumor foci, Maximum diameter of primary tumor foci.

TyG Model 5 (Extracapsular extension): adjusted for age, gender, TSH, BMI,TC, LDL-C, HDL-C, uric acid, Number of primary tumor foci, Distribution of tumor foci, Maximum diameter of primary tumor foci

FBG Model 5 (Lymph node metastasis): adjusted for age, gender, TSH, BMI, TG, TC, LDL-C, HDL-C, uric acid, Number of primary tumor foci, Distribution of tumor foci, Maximum diameter of primary tumor foci, Extracapsular extension.

### Incremental model performance of FBG and TyG

For extracapsular extension, the AUCs of the baseline, FBG, and TyG models were 0.710 (95% CI: 0.675-0.746), 0.738 (95% CI: 0.703-0.772), and 0.722 (95% CI: 0.686-0.757), respectively. For lymph node metastasis, the corresponding AUCs were 0.712 (95% CI: 0.682-0.743), 0.727 (95% CI: 0.697-0.757), and 0.714 (95% CI: 0.683-0.745), respectively (**Figure 1**; **Supplementary Table 2**).

**Figure 1:**
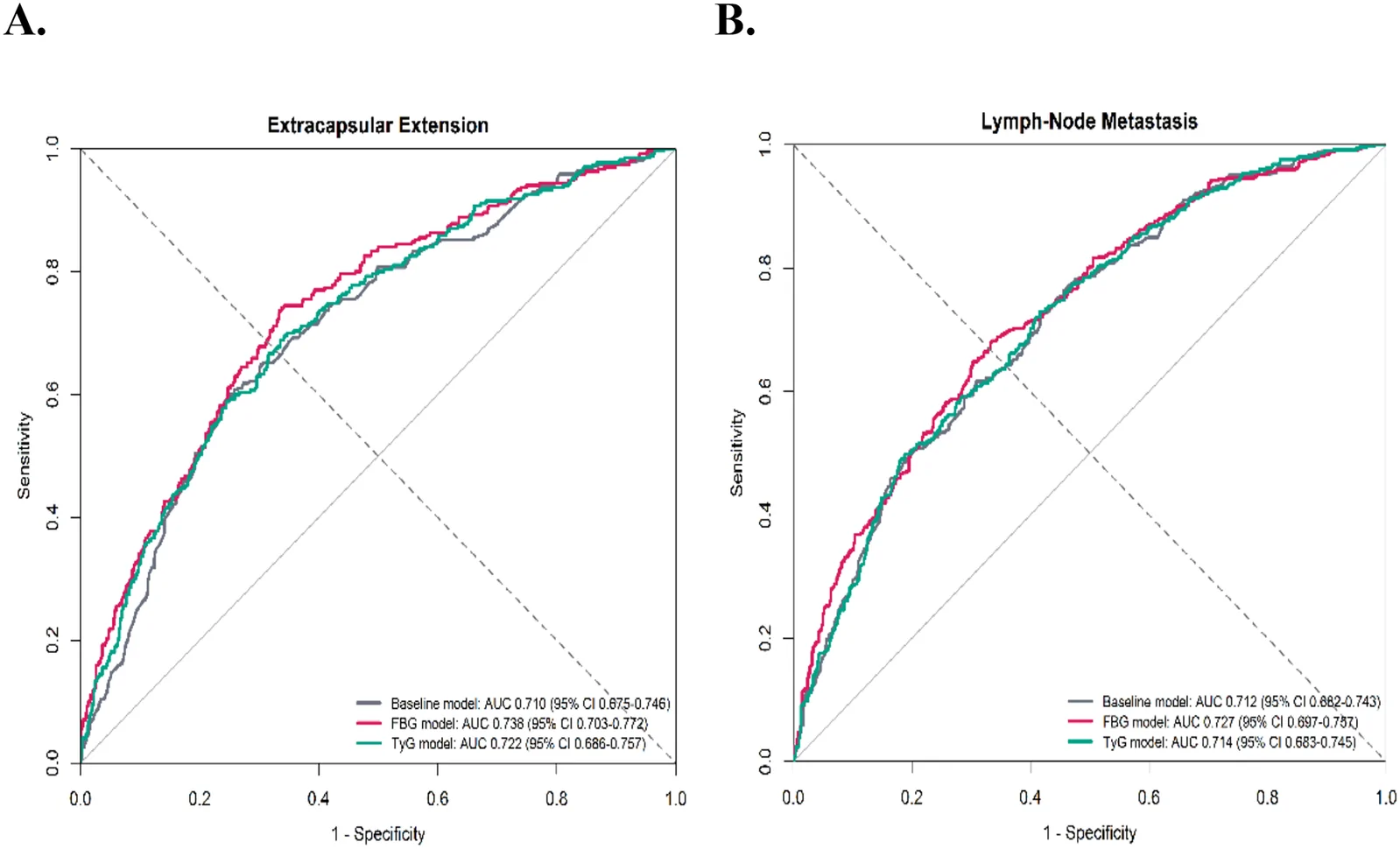
**(A)** Models for extracapsular extension. The baseline model included age, sex, thyroid-stimulating hormone (TSH), body mass index (BMI), total cholesterol (TC), low-density lipoprotein cholesterol (LDL-C), high-density lipoprotein cholesterol (HDL-C), uric acid, primary tumor number and distribution, and maximum primary tumor diameter. The FBG model comprised the baseline model plus fasting FBG, whereas the TyG model comprised the baseline model plus the TyG index. **(B)** Models for lymph node metastasis. The baseline model included age, sex, TSH, BMI, TC, LDL-C, HDL-C, uric acid, primary tumor number and distribution, maximum primary tumor diameter, and extrathyroidal extension. The FBG and TyG models were constructed by adding FBG and the TyG index to the baseline model, respectively.

Compared with the baseline model, the addition of FBG significantly improved the AUC for both extracapsular extension and lymph node metastasis, with AUC differences of 0.028 (P = 0.003) and 0.015 (P = 0.015), respectively. The addition of TyG increased the AUCs for extracapsular extension and lymph node metastasis by 0.012 (P = 0.104) and 0.002 (P = 0.396), respectively (**Supplementary Table 3**).

For extracapsular extension, the addition of FBG yielded a continuous NRI of 0.406 (95% CI: 0.242-0.561, P = 0.002) and an IDI of 0.040 (95% CI: 0.019-0.068, P = 0.002). The addition of TyG yielded a continuous NRI of 0.285 (95% CI: 0.114-0.414, P = 0.004) and an IDI of 0.015 (95% CI: 0.004-0.033, P = 0.002). For lymph node metastasis, the addition of FBG significantly improved the IDI (IDI = 0.020, 95% CI: 0.007-0.037, P = 0.002). The NRI (0.016, *P* = 0.525) and IDI (0.002, *P* = 0.034) of TyG showed no obvious incremental contribution. (**Supplementary Table 4**).

After internal validation with 1000 bootstrap resamples, the optimism-corrected AUCs of the FBG model for extracapsular extension and lymph node metastasis were 0.722 (95% CI: 0.697-0.765) and 0.712 (95% CI: 0.691-0.748), respectively. The corresponding corrected AUCs of the TyG model were 0.704 (95% CI: 0.679-0.747) and 0.698 (95% CI: 0.677-0.736). Mean optimism in the AUC ranged from 0.015 to 0.018. Optimism-corrected Brier scores ranged from 0.169 to 0.222, and calibration slopes ranged from 0.881 to 0.902 (**Supplementary Table 5**).

## Discussion

In this study, we presented findings from a retrospective study of a large cohort of PTC patients, showing that elevated FBG, TG, UA, BMI and TyG were associated with adverse clinicopathological characteristics. Further analyses showed that elevated FBG was associated with extracapsular extension and lymph node metastasis, whereas elevated TyG was associated with extracapsular extension.

It has also been reported that hyperglycemia and obesity may promote the development of PTC and may be associated with larger primary foci size, extracapsular extension, lymph node metastasis, and advanced TNM stage (22). However, other studies found no correlation with PTC clinicopathological features, tumor metastasis, or recurrence of PTC (23). Therefore, the relationship between metabolic indicators and pathological characteristics remains elusive.

This study demonstrates that PTC patients with DM not only have significant metabolic disturbances, including dysregulated glucose-lipid metabolism and obesity, but also show more aggressive tumor behavior, as evidenced by higher proportions of tumor diameter >1 cm, extracapsular extension, lymph node metastasis, intermediate-to-high recurrence risk, and advanced TNM stage. These findings suggest a possible association between metabolic abnormalities and PTC progression. After adjusting for potential factors including sex, age, and TSH, multivariate logistic regression analysis demonstrated that elevated FBG, TG, UA, and BMI were associated with an increased risk of aggressive pathological features. Specifically, the hyperglycemia group (FBG≥ 6.1 mmol/L) and the high BMI group (≥25 kg/m2) had an increased risk of multifocal primary tumor foci, maximum diameter of foci >1 cm, extracapsular extension, lymph node metastasis, and intermediate-to-high risk stratification for recurrence. Collectively, these results indicate that elevated BMI and metabolic abnormalities are associated with adverse pathological features.

The mechanisms by which obesity and metabolic abnormalities promote PTC progression are complex. A combination of factors, including insulin resistance, TSH level, inflammatory cytokines, adipokines, and alterations in estrogen, has been implicated in PTC pathogenesis (24). First, obesity and related metabolic abnormalities can induce IR and hyperinsulinemia, thereby enhancing the systemic effects of insulin-like growth factors (IGFs). These potent growth-promoting factors may stimulate mitogenic cascades through activation of the mTOR pathway in thyroid cancer cells, promoting angiogenesis and tumor proliferation (24). In addition, obesity and metabolic dysregulation induce a state of low-grade chronic inflammation that may accelerate cancer growth by increasing the release of inflammatory mediators (25). Leptin and its receptor (Ob-R) are overexpressed in PTC associated with obesity and promote proliferation of PTC cell lines through the PI3K/AKT pathway (13). Leptin may also enhance resistance to apoptosis and angiogenesis through phosphorylation of AKT and ERK (26) and can stimulate macrophages to release proinflammatory cytokines, including interleukin IL-1, IL-6, and tumor necrosis factor-α (TNF-α) (27). IL-6 may promote thyroid cancer growth and dissemination through the JAK/STAT3 pathway (28), whereas TNF-α can induce granulocyte colony-stimulating factor production by thyroid fibroblasts, thereby contributing to thyroid cancer progression (29). Conversely, reduced or absent adiponectin expression may increase vascular endothelial growth factor expression and impair tumor-suppressive activity through c-Jun N-terminal kinase and STAT3 signaling, favoring a more invasive phenotype (14). Elevated TSH can directly stimulate PTC cell proliferation (12), and increased estrogen signaling may also contribute to PTC pathogenesis (3). Together, these mediators can promote PTC proliferation, invasion, and metastasis through multiple signaling pathways and target genes, including PI3K/AKT, JAK2-STAT3, RAS-BRAF, mTOR, and AMP-activated protein kinase (24). Thus, the association between obesity, metabolic abnormalities, and PTC progression likely reflects a complex interplay among inflammation, metabolic dysfunction, and hormonal signaling rather than a single pathway.

As a central part of the metabolic syndrome, IR participates in the pivotal pathological mechanism through which abnormalities in glucose and lipid metabolism and obesity contribute to the progression of PTC. The TyG index offers high sensitivity and specificity for IR assessment and is considered a valuable predictor and novel surrogate marker across different populations (30). The TyG index correlates more strongly with hyperglycemic clamp results than HOMA-IR, likely because it captures both hepatic and muscular insulin resistance (31). Previous studies have linked the TyG index to cancer risk, suggesting that it may capture metabolic disturbances involved in tumor development (18). A population-based study in the United States reported that elevated TyG was associated not only with the prevalence of gastrointestinal cancers but also with cancer prognosis (32). In the present study, patients in the highest TyG quartile had a greater risk of extracapsular extension, supporting an association between TyG and PTC aggressiveness. Although the precise role of TyG in cancer remains uncertain, proposed mechanisms center on IR and hyperinsulinemia. Elevated TyG reflects a vicious cycle of glucolipotoxicity that can promote systemic oxidative stress through excessive free-radical generation. Triglyceride accumulation increases circulating free fatty acids; together with glucose, these substrates can activate nuclear factor-κB, increase nitric oxide and reactive oxygen species production, and stimulate inflammatory cytokines. The resulting oxidative stress can impair insulin signaling and aggravate IR (33).

Although the overall prognosis of PTC is favorable, some tumors display highly aggressive features, including larger size, multifocality, extracapsular extension, and cervical lymph node metastasis. These characteristics enable local invasion and distant dissemination and are associated with recurrence and unfavorable outcomes. ECE and cervical LNM are central pathological considerations in determining the extent of thyroidectomy and cervical lymph node dissection. They are also strong independent predictors of local recurrence and directly inform assessments of TNM stage and American Thyroid Association recurrence risk, thereby guiding decisions regarding radioactive iodine therapy and the intensity of TSH suppression. Accordingly, ECE and LNM were selected as the primary outcomes for multivariable association analyses and ROC-based prediction. After adjustment for age, sex, TSH, BMI, TG, TC, LDL-C, HDL-C, and UA, elevated FBG (≥6.1 mmol/L) was associated with extracapsular extension and lymph node metastasis (ORs=2.965 and 2.516, respectively). After adjustment for age, sex, TSH, BMI, TC, LDL-C, HDL-C, and UA, elevated TyG was associated with extracapsular extension (OR = 2.296). These associations persisted after further adjustment for pathological factors, including the number and distribution of tumor foci and maximum primary tumor diameter, with additional adjustment for extracapsular extension in the lymph node metastasis model: elevated FBG remained associated with extracapsular extension (OR = 2.778) and lymph node metastasis (OR = 2.034), whereas elevated TyG remained associated with extracapsular extension (OR = 2.337). These findings suggest that hyperglycemia and IR are associated with aggressive pathological features of PTC.

This study further evaluated the incremental value of FBG and TyG. The baseline model included age, sex, TSH, BMI, total cholesterol, low-density lipoprotein cholesterol, high-density lipoprotein cholesterol, uric acid, tumor number and distribution, and maximum primary tumor diameter. For extrathyroidal extension, adding FBG or TyG to the baseline model provided incremental value, with NRI values of 0.406 and 0.285 and IDI values of 0.040 and 0.015, respectively. For lymph node metastasis, the addition of FBG resulted in a significant IDI, whereas the NRI was not significant. The NRI and IDI of TyG showed no obvious incremental contribution.

This study has some limitations. First, it was a single-center, retrospective study. Indicators of glucose and lipid metabolism, uric acid, and BMI were collected at baseline; therefore, a temporal relationship between metabolic abnormalities and clinicopathological features could not be established. In addition, smoking, alcohol consumption, glucose-lowering medications, glycated hemoglobin, and inflammatory markers could not be included because of missing retrospective data, raising the possibility of residual confounding. Second, although TyG is considered a surrogate marker of IR, insulin levels, the homeostatic model assessment of insulin resistance, and longitudinal metabolic changes were not directly assessed. Therefore, the strength of the correlations between TyG and these measures could not be assessed. Finally, the findings have not been externally validated. Large multicenter prospective cohort studies, mechanistic investigations, and external validation are therefore needed to clarify the causal relationships between metabolic indicators and PTC and to determine whether metabolic interventions can influence PTC progression and prognosis.

## Conclusion

In conclusion, elevated FBG, TG, UA, BMI, and TyG were associated with adverse pathological features of PTC. FBG was associated with extracapsular extension and lymph node metastasis and improved the risk reclassification for both, whereas TyG was associated with extracapsular extension but showed no clear incremental value for risk reclassification of lymph node metastasis.

## Data Availability

The original contributions presented in the study are included in the article/**Supplementary Material**, further inquiries can be directed to the corresponding author/s.
