# Supplementary material for: Associations of fasting blood glucose and the triglyceride–glucose index with aggressive pathological features of papillary thyroid carcinoma
Source: Front Endocrinol (Lausanne). 2026 Oct 21;17:1931124. doi: 10.3389/fendo.2026.1931124 (PMC13730869; doi:10.3389/fendo.2026.1931124)
Supplement: Supplementary file 1 [file Supplementaryfile1.docx]

**Associations of Fasting Blood Glucose and the Triglyceride–Glucose (TyG) Index With Aggressive Pathological Features of Papillary Thyroid Carcinoma (PTC)**

Xiang Xu 12, Kun Gong 3, Chengqian Li 2, Yonglu Hu 2, Cong Liu 24, Pei Guo 5, Wenjuan Zhao 2, Yangang Wang 2 $, Bingzi Dong 2 $

1 Department of Cardiology, The Affiliated Hospital of Qingdao University, Qingdao 266003, China

2 Department of Endocrinology and Metabolism, The Affiliated Hospital of Qingdao University, Qingdao 266003, China.

3 Department of Echocardiography, The Affiliated Hospital of Qingdao University, Qingdao 266003, China.

4 Department of Surgical Intensive Care Unit, Yantai Yantaishan Hospital, Yantai 264000, China

5 Medical Records Management Center, The Affiliated Hospital of Qingdao University, Qingdao 266003, China

Corresponding author:

Prof. Bingzi Dong

Department of Endocrinology and Metabolism, The Affiliated Hospital of Qingdao University, Qingdao 266003, China.

Email: dongbingzi@hotmail.com; dongbingzi@qdu.edu.cn

ORCID: 0000-0003-2446-0725

Co-Corresponding author:

Prof. Yangang Wang

Department of Endocrinology and Metabolism, The Affiliated Hospital of Qingdao University, Qingdao 266003, China.

Email: [wangyg1966@126.com](mailto:wangyg1966@126.com); wangyg@qdu.edu.cn

ORCID：0000-0002-4653-8899

**Suppl Table 1. Clinicopathological characteristics, metabolic indicators and thyroid function levels of PTC patients.**

| **Variable** | **Total (%)** |
| --- | --- |
| Gender | |
| Male | 244(23.51) |
| Female | 794(76.49) |
| Age(year) | |
| <55 | 785(75.63) |
| ≥55 | 253(24.37) |
| Maximum diameter of primary tumor foci |  |
| ≤1 cm | 623(60.02) |
| >1cm | 415(39.98) |
| Number of primary tumor foci |  |
| Unifocal | 686(66.09) |
| Multifocal | 352(33.91) |
| Localization of tumor foci |  |
| Unilateral | 774(74.57) |
| Bilateral | 264(25.43) |
| Extracapsular extension |  |
| Yes | 270(26.01) |
| No | 768(73.99) |
| Lymph node metastasis | |
| Yes | 500(48.17) |
| No | 538(51.83) |
| Recurrence risk stratification | |
| Low-risk | 440(42.38) |
| Intermediate-high risk | 598(57.61) |
| TNM stage | |
| I-II | 975(93.93) |
| III-IV | 63(6.06) |
| Diabetes mellitus | |
| Yes | 102(9.83%) |
| No | 936(90.17%) |
| FBG (mmol/L) | |
| ＜6.1 | 848 (81.70) |
| ≥6.1 | 190 (18.30) |
| TG (mmol/L) | |
| ≤1.92 | 873 (84.10) |
| >1.92 | 165 (15.90) |
| TC (mmol/L) | |
| ≤5.62 | 764 (73.60) |
| >5.62 | 274 (26.40) |
| LDL-C(mmol/L) | |
| ≤3.12 | 677 (65.22) |
| >3.12 | 361 (34.78) |
| HDL-C(mmol/L) | |
| <0.8 | 6(0.58) |
| ≥0.8 | 1032 (99.42) |
| BMI (kg/m2) | |
| <25 | 523 (50.39) |
| ≥25 | 515 (49.61) |
| UA (umol/L) | |
| ≤420 | 965 (92.97) |
| >420 | 73 (7.03) |
| TyG | |
| ＜9.2 | 903 (86.99) |
| ≥9.2 | 135 (13.01) |
| TSH (mIU/L) |  |
| ≤4.2 | 1029 (99.13) |
| >4.2 | 9 (0.87) |

FBG, TG, TC, LDL-C, UA, TyG are grouped based on the upper limit of normal. HDL-C is grouped based on the lower limit of normal. BMI is grouped based on WHO standards.

**Suppl Table 2. ROC analyses of the baseline, FBG, and TyG models for extracapsular extension and lymph node metastasis.**

| **Outcome** | **Model** | **AUC (95% CI)** | **Probability threshold** | **Youden index** | **Sensitivity, % (95% CI)** | **Specificity, % (95% CI)** |
| --- | --- | --- | --- | --- | --- | --- |
| Extracapsular Extension | baseline model | 0.710 (0.675-0.746) | 0.311 | 0.354 | 60.0 (55.6-78.2) | 75.4 (56.9-78.5) |
| Extracapsular Extension | FBG model | 0.738 (0.703-0.772) | 0.239 | 0.404 | 74.1 (64.1-79.6) | 66.3 (62.5-74.9) |
| Extracapsular Extension | TyG model | 0.722 (0.686-0.757) | 0.251 | 0.354 | 69.6 (55.9-78.1) | 65.8 (57.3-78.5) |
| Lymph-Node Metastasis | baseline model | 0.712 (0.682-0.743) | 0.412 | 0.309 | 76.4 (46.6-80.8) | 54.5 (51.3-84.8) |
| Lymph-Node Metastasis | FBG model | 0.727 (0.697-0.757) | 0.469 | 0.347 | 68.2 (56.8-81.0) | 66.5 (52.6-77.1) |
| Lymph-Node Metastasis | TyG model | 0.714 (0.683-0.745) | 0.435 | 0.314 | 73.0 (47.4-80.6) | 58.4 (50.9-84.4) |

**Suppl Table 3. Incremental changes in the area under the receiver operating characteristic curve and pairwise comparisons using DeLong's test.**

| **Outcome** | **Comparison** | **Delta AUC** | **P** |
| --- | --- | --- | --- |
| Extracapsular Extension | FBG model vs baseline model | 0.028 | 0.003 |
| Extracapsular Extension | TyG model vs baseline model | 0.012 | 0.104 |
| Lymph-Node Metastasis | FBG model vs baseline model | 0.015 | 0.015 |
| Lymph-Node Metastasis | TyG model vs baseline model | 0.002 | 0.396 |

**Suppl Table 4. NRI and IDI for the FBG and TyG models compared with the baseline model.**

| **Outcome** | **Comparison** | **NRI (95% CI)** | **P** | **IDI (95% CI)** | **P** |
| --- | --- | --- | --- | --- | --- |
| Extracapsular Extension | FBG model vs baseline model | 0.406 (0.242-0.561) | 0.002 | 0.040 (0.019-0.068) | 0.002 |
| Extracapsular Extension | TyG model vs baseline model | 0.285 (0.114-0.414) | 0.004 | 0.015 (0.004-0.033) | 0.002 |
| Lymph-Node Metastasis | FBG model vs baseline model | 0.162 (-0.003-0.281) | 0.056 | 0.020 (0.007-0.037) | 0.002 |
| Lymph-Node Metastasis | TyG model vs baseline model | 0.016 (-0.074-0.166) | 0.525 | 0.002 (0.000-0.009) | 0.034 |

**Suppl Table 5. Bootstrap estimates of discrimination and calibration for the FBG and TyG models.**

| **Outcome** | **Model** | **Apparent AUC** | **Mean AUC optimism** | **Corrected AUC (95% CI)** | **Brier: apparent / corrected** | **Calibration slope: apparent / corrected** |
| --- | --- | --- | --- | --- | --- | --- |
| Extracapsular Extension | FBG model | 0.738 | 0.016 | 0.722 (0.697-0.765) | 0.165 / 0.169 | 1.000 / 0.895 |
| Extracapsular Extension | TyG model | 0.722 | 0.018 | 0.704 (0.679-0.747) | 0.169 / 0.174 | 1.000 / 0.881 |
| Lymph-Node Metastasis | FBG model | 0.727 | 0.015 | 0.712 (0.691-0.748) | 0.211 / 0.217 | 1.000 / 0.902 |
| Lymph-Node Metastasis | TyG model | 0.714 | 0.016 | 0.698 (0.677-0.736) | 0.215 / 0.222 | 1.000 / 0.894 |
